# A Numerical Investigation of Graphene-Based Hilbert-Shaped Multi-Band MIMO Antenna for the Terahertz Spectrum Applications

**DOI:** 10.3390/s23010037

**Published:** 2022-12-20

**Authors:** Khaled Aliqab, Meshari Alsharari, Vishal Sorathiya, Ammar Armghan

**Affiliations:** 1 Department of Electrical Engineering, College of Engineering, Jouf University, Sakaka 72388, Saudi Arabia; 2 Faculty of Engineering and Technology, Parul Institute of Engineering and Technology, Parul University, Waghodia Road, Vadodara 391760, India

**Keywords:** terahertz, MIMO, ECC, TARC, mllimetre-wave, gain, bandwidth

## Abstract

We proposed the numerical investigation of Hilbert-shaped multiple-input multi-output (MIMO) with multi-band operation characteristics using graphene resonator material, which operates on the band of 1 to 30 THz of the frequency range. This numerical investigation of antenna structure was carried out for the multiple antenna types, consisting of graphene as a regular patch, Hilbert order 1, and Hilbert order 2 designs. This antenna is investigated for the multiple physical parameters, such as return loss, gain, bandwidth, radiation response, Envelope Correlation Coefficient (ECC), Total Active Reflection Coefficient (TARC), Mean Effective Gain (MEG), Directivity Gain (DG), and Channel Capacity Loss (CCL). These variables are also determined to verify compatibility and the difficulties connected with communicating over a short distance. The THz MIMO antenna that was recommended offers strong isolation values in addition to an operational band. The maximum gain of ~10 dBi for the band of <15 THz and ~17 dBi for the band of the >15 THz frequency range of the proposed antenna structures. The proposed antennas are primarily operated in three bands over 1 to 30 THz of frequency. This work aims to create a brand new terahertz antenna structure capable of providing an extraordinarily wider bandwidth and high gain while keeping a typical compact antenna size suited for terahertz applications.

## 1. Introduction

The technology behind wireless communication saw a significant transformation over the past several decades. It is mostly due to the growing need for faster data rates in the modern trend of wireless communication [1]. Because of the research achievements in mobile communication systems that permitted enhanced data connection [2,3], these advancements resulted in various new application possibilities. A variety of institutions are carrying out this research. However, several factors must be considered, including a substantial amount of propagation route loss and a restricted communication distance. THz communications offer an exciting promise, but also important obstacles. We must not lose sight of the fact that the features of the THz antenna directly impact the performance of THz systems. It is something that must not be forgotten. The small size and great sensitivity of antennas for transmitting and sensing THz waves are two reasons for their huge demand. Broadcasting, satellite, and mobile communications, the identification of explosives and weapons (including the development of explosives and weapons identification systems), multimedia, environmental sensing (including the development of radars), and medical systems are some of the many applications that THz devices can be used for [4]. Wireless communication technology saw major changes in the last several decades due to the growing need for higher data rates in the current trend. The IEEE’s 0.3 to 10 THz terahertz wireless communications (TeraCom) standard might be the future wireless technology that fulfils the requirements of the Tbps datarate and massive channel capacity [5]. Terahertz wireless communications (TeraCom) that take advantage of the greater bandwidth that can be unlocked and used for future large-scale civic and commercial applications. Scientists are conducting a large amount of research on terahertz communication at shorter wavelengths. Over the last several decades, a substantial amount of research was conducted, and technological advancements were made in this area due to the vast number of potential applications. As a consequence of this, there is a growing need for upgraded antennas that are capable of functioning at terahertz (THz) frequencies [4]. It is possible to build tiny, high-performance antennas capable of sending and receiving signals in the terahertz frequency ranges. This helps the general development of more advanced terahertz communication systems in the future. The THz bands suffer from a severe lack of detectors and sources, which negatively impacts the performance of devices that operate in the terahertz domain. For the higher frequency bands, such as the THz band, we have access to more channel capacity and increase the route loss and the sensitivity to blocking events. This is because higher frequency bands have a smaller bandwidth than lower frequency bands. As a direct result, there is a growing demand for more compact antennas with high gain, high efficiency, and expanded operational bandwidth to combat these problems. As a direct consequence of the demand for ultrahigh performance characteristic parameters for micro- and nano-scaled THz antennas, many fresh problems and possibilities became available. These new challenges and opportunities undoubtedly contribute to antenna technology’s expansion. There are many different antenna designs, such as metallic [6], horn [7], lens [8], metamaterial [9,10,11], on-chip [12], dielectric [13,14], and leaky-wave antenna [15,16], photoconductive [17], yagi antenna [18], log periodic antenna [19], cuboidal [20], and slotted antenna [21], that were described in literature. For these antennas to perform their intended role, they must have a bigger physical footprint and a more involved design process. Incorporating planar electronics into these THz antenna designs is another challenge that must be overcome. Microstrip antennas, for their part, are experiencing a rise in popularity as planar technology continues to become more widespread. Terahertz short-range wireless applications are particularly well suited to these devices due to the many benefits, such as low cost, simplicity of design, lightweight, and tiny size. However, despite its many benefits, it has a narrow bandwidth, which prevents it from being utilized at high THz frequencies. To circumvent this challenge and enable a wide range of applications to be carried out in the THz frequency domain, small antennas that weigh very little and have a broad operating bandwidth are now under development. Researchers built a variety of small THz antennas and reported their findings in the relevant academic literature. However, because they are small, they can only function within extremely narrow bandwidths [22,23,24]. In addition, a study and a suggestion for designing a multi-layer array antenna are presented in reference [25]. The operational bandwidth of the THz antenna topologies discussed in this article were extended to compensate for the larger antenna diameter. Researchers who published their findings in publications reviewed by their peers designed wideband THz antennas to resolve the fading problem in high-speed, short-range wireless applications that operate at high frequencies in the THz spectrum. These researchers published their findings in publications. In wireless networks, utilizing MIMO antenna systems may help reduce the risks associated with signal fading.

In this paper, we designed and numerically investigated (simulation study) the Hilbert-shaped MIMO patch antenna with different design configurations for the 1–30 THz frequency band. We calculated the different physical parameters of the proposed antenna, such as S parameters, TARC, ECC, CCL, DG, MEG, and polar plots. We calculated the effect of the Hilbert shape over the different orders of its shape. We also discussed the influence of the Hilbert shape over the standard patch antenna structure. Because this design achieves potentially beneficial outcomes across all antenna characteristics, it is well-suited for use in THz wireless communication settings.

The highlights and novelties of the proposed graphene-based Hilbert-shaped antenna are as follows:

- The microstrip technique was utilized to design the graphene-based Hilbert-shaped MIMO antenna. Compared to other THz antennas, this one is more advantageous in size, weight, ease of construction, and cost.
- The proposed antenna has a significantly large bandwidth of 8.1 THz.
- The MIMO structure that was developed provides improved results across various performance criteria.
- This antenna helps overcome the challenges associated with short-distance communication, such as increased interference, signal fading, and multipath propagation. CCL < 0.5 bps/Hz/s, ECC < 0.01, MEG ≤ −3.0 dB, TARC ≤ −10.0 dB, and DG ≈ 10 dB are all parameters that help to achieve this.

## 2. Hilbert Shape Design and Numerical Parameters

**Figure 1:**
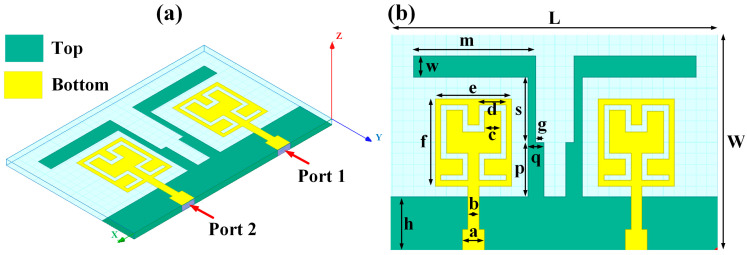
Schematic view of the Hilbert patch-shaped MIMO antenna. (**a**) A view of the antenna in three dimensions, and (**b**) views of the antenna from the top and bottom.

A schematic of the Hilbert-shaped MIMO antenna with order 2 is shown in Figure 1. Figure 1a shows the three-dimensional (3D) view of the MIMO antenna, and Figure 1b shows the top and bottom view of the antenna. The dimensions of the structures are shown in Table 1. To determine the overall behaviour of the proposed antenna, we determined that there are a total of five distinct designs. These designs and their descriptions are shown in Table 2. Graphene material of 0.03 m was used to build the antenna geometry. With a loss tangent of 0.004, the graphene sheet’s conductivity was fixed at 10^8^ S/m. Graphene is widely used in a variety of terahertz (THz) applications because of its outstanding electromagnetic, mechanical, and tunable characteristics [26,27]. It was also noted that the multilayered graphene ink/powder does not have an extensive range of tuning options, as several papers pointed out. Thus, the entire structure is based on constant conductivity. Polyimide material is chosen as the substrate for the proposed antenna design. The MIMO antenna is designed using the formula mentioned in [20] to calculate the minimum and maximum length of the dipole elements. The values of the minimum and maximum dipole elements are calculated using $L_{min}=\frac{c}{f_{max}\sqrt{\varepsilon_{r}}}$ and $L_{max}=\frac{c}{f_{min}\sqrt{\varepsilon_{r}}}$. Here, $f_{max},\;f_{min},\;\;\varepsilon_{r}\;\mathrm{and}\;c\;$is the maximum frequency, minimum frequency, permittivity of the substrate, and velocity of light, respectively. We determined that the dipole element’s lowest and maximum lengths are 7.2 µm and 144 µm, respectively. Based on this calculation, the total patch antenna size was determined to be 2t = 140 nm. However, electromagnetic waves are not restricted only to the substrate in this construction. The realistic permittivity estimate must thus be recalculated for the total building dimensions. The microstrip line formula $\varepsilon_{eff}=\frac{\varepsilon_{r}+1}{2}+\frac{\varepsilon_{r}-1}{2}{\left[1+12\frac{h}{l}\right]}^{-1/2}$ is used to identify the effective permittivity of the structure. There are two variables in this equation: h and l, representing the substrate’s thickness and feed line length. The approximate calculated $\varepsilon_{eff}$ value is 12.22. The overall structure size is calculated by $L=\frac{\lambda_{max}}{4}\left(1-\frac{1}{B_{s}}\right)\cot\alpha$. An estimated 8 dB increase may be achieved by using the appropriate bandwidth (*Bs*) and the 12.132° angle. The obtained length is 310 µm in total. Single-antenna radiation uses these design requirements. The separation between the two MIMO components raises the overall size to 620 × 400 µm^2^. The proposed antenna can be fabricated using CNC milling, laser imprint machines, and 3D printer setups. The antenna of mmWave can be measured using the available measurement setup using Keysight N5245PNA-X network analyzer, which provides a measurement capacity of up to 1.1 THz [28].

## 3. Results and Discussion

**Figure 2:**
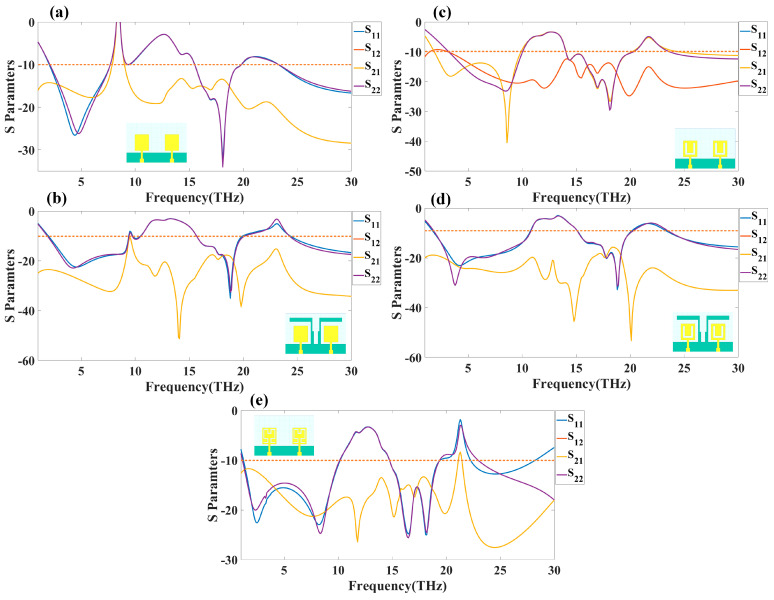
Calculated return loss (S parameters) for the MIMO antennas. S parameters response for (**a**) simple patch antenna with the rectangular back side (Design 1), (**b**) Simple patch antenna with modified back side with covered sides (Design 2), (**c**) Hilbert shape order 1 with the rectangular back side (Design 3), (**d**) Hilbert shape order 1 with modified back side with covered sides (Design 4), and (**e**) Hilbert shape order 2 with the rectangular back side (Design 5). Orange line is defined at −10 dB point to measure the reference values of S_11_ and S_21_.

The HFSS antenna modelling environment was used to build the antenna construction. Subtracting the required area from a rectangular antenna patch produces the patterned Hilbert shape. Graphene is used for the top and ground layers, making them conductive. Since air is considered a substance, the radiation box is positioned around the antenna. For the outer radiation box, we specify the radiation border condition. The ports depicted in Figure 1 are energized as a single, combined port by assuming the ground as a reference plane. The inputs are treated as a single excitation lumped port source, and the standard HFSS port conditions are used. The simulation is run. The directivity outcomes are produced by varying the port excitation settings using a frequency sweep on the exciting port. The HFSS-simulated antenna’s reflectance loss, gain, and directivity values can be calculated using pre-processing methods. The computed differences in the S parameters between the Design 1 structure and the Design 2 structure are depicted in Figure 2a,b. The three-operating band for Design 1 is shown in Figure 2a. We considered the values of the S parameters below −10 dB for comparative analysis in all cases. The maximum bandwidth obtained in Design 1 is 6.3 THz at the minimum return loss of −16.08 dB. The minimum return loss of the −39.05 dB for the bandwidth of 4.2 THz is also observed.

Similarly, in Design 2, the minimum return loss is observed as −38.09 dB in the operating bandwidth of 3.2 THz. As shown in Figure 2b, the most significant bandwidth measured came in at 7.4 THz for the first band of operation. The detailed comparison between all the designs in terms of the operating band, minimum and maximum frequency, minimum return loss, and bandwidth is shown in Table 3. Table 3 was prepared with the modified results by considering the <−10 dB of the return loss in S_11_ and S_22_ parameters. The observation presented in Table 3 also considers that the other return loss, such as S_21_ and S_12_, must be <−10 dB. In this observation of the band, $f_{min}$, $f_{max}$, and bandwidth, all S parameters are considered as <−10 dB.

Figure 2c–e shows the derived S parameters responsible for designs 3–5. In Design 3, the full resonating band is observed between a 14.1 and 20 THz frequency with a bandwidth of 5.9 THz and return loss of −29.57 dB, as shown in Figure 2c. Figure 2d shows the variation in S parameters for Design 4 with a maximum operating band of 6.9 THz between a 3.6 and 10.5 THz frequency range with −29.78 dB of return loss. Design 5 (Figure 2e) generates the full resonating band of 8.8 THz between a 1.3 and 10.1 THz frequency range with −24.7 dB of the return loss. Overall, the maximum bandwidth of 8. 8 THz is observed in Design 5, while the minimum return loss of −40.46 dB is observed in Design 3. It is also observed that the change in the shape of the patch from regular to Hilbert also allows us to change the antenna operating conditions. The shape of the Hilbert order and back side geometry. The different order of the Hilbert also allows us to change the operating frequency and band of the overall structure. We can observe the overlapping return loss values in Figure 2 for the Design 1, 2, and 4 (Figure 2a,b,d). Similarly, In Figure 2c,e, some of the frequency bands are overlapping, while some are not. The discrepancy in this graph is due to the tetrahedral meshing conditions, where the subtracted part of the Hilbert shapes are covered with different densities of the tetrahedral conditions. In the case of the individual port excitation, the meshing from left to right and right to left is different. A more minor variation in the return loss can also be observed in Figure 2a,c,d near the 5 THz frequency. This variation is higher in Figure 2c,e at lower and higher THz frequency bands. The validity of the results where the interference of S parameters in cross-port conditions such as S12 and S21 overlap in all the antennae except Figure 2c is demonstrated by Design 3. As an overall observation, we have the S parameters values < −10 dB in the entire band (3 to 10 THz) in Design 3, which will give us an idea of effective radiation.

## 4. MIMO Antenna Parameters

In Equation (1), the electric field magnitude is used to determine ECC; *i*th and *j*th denote the solid angle components of ECC. Evaluating ECC using far-field radiation parameters takes a long time and much effort. Another method described in [29] for finding ECC using the S-parameters approach is shown in Equation (2).

(1) $$\rho_{ij}={\left|\frac{\iint^{\;}{\left(E_{\theta_{i}}\cdot E_{\theta_{j}}^{*}+E_{\varphi }:E_{\varphi_{j}}^{*}\right)}^{d\Omega }}{\iint^{\;}\left(E_{\theta_{i}}\cdot E_{\theta_{i}}^{*}+E_{\varphi_{i}}\cdot E_{\varphi_{i}}^{*}\right)d\Omega \iint^{\;}{\left(E_{\theta_{j}}\cdot E_{\theta_{j}}^{*}+E_{\varphi_{j}}\cdot E_{\varphi_{i}}^{*}\right)}^{d\Omega }}\right|}^{2}$$

(2) $$\rho_{ij}=\frac{{\left|S_{11}^{*}S_{12}+S_{21}^{*}S_{22}\right|}^{2}}{\left(1-\left({\left|S_{22}\right|}^{2}+{\left|S_{12}\right|}^{2}\right)\right)\left(1-\left({\left|S_{11}\right|}^{2}+{\left|S_{21}\right|}^{2}\right)\right)}$$

**Figure 3:**
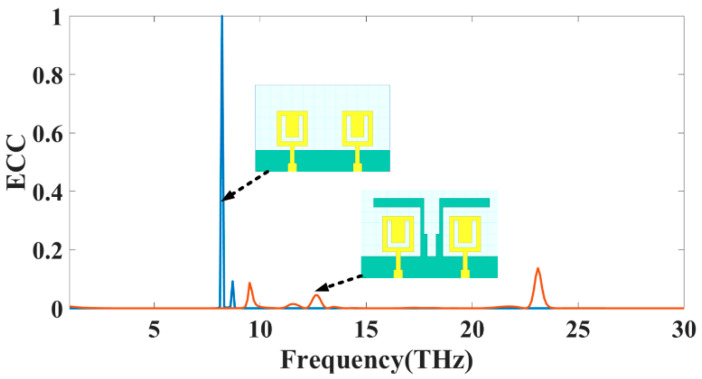
The simulated value of the ECC for MIMO structure Design 3 and Design 4.

Figure 3 presents the calculations made about the ECC for antennas of types 3 and 4. In Table 2, it can be seen that the antenna’s ECC is less than 0.001 over its primary operating band. Because of these ECC levels, the system has greater stability. The lower the ECC value, the fewer connections there are between the various components of the antenna. Since the values are so low, the MIMO performance of the antenna is assumed to be very good. The fading effects can be mitigated by combining antenna components with distinct fading characteristics.

(3) $$\mathrm{DG}=10\sqrt{1-|\mathrm{ECC}{|}^{2}}$$

(4) $${\mathrm{MEG}}_{i}=0.5\eta_{i,\;\mathrm{rad}}=0.5\left[1-\overset{M}{\underset{j=1}{\sum }}{\left|S_{ij}\right|}^{2}\right]$$

**Figure 4:**
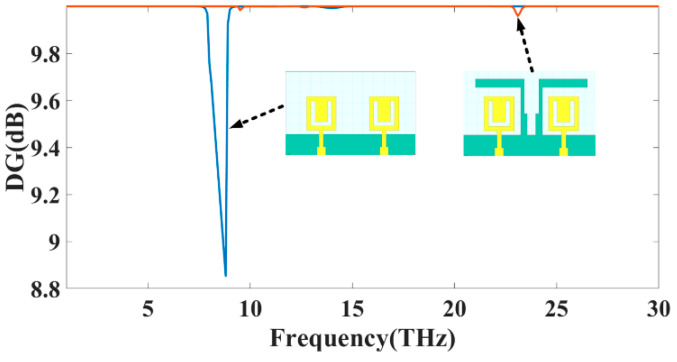
The DG (dB) simulated value for MIMO structure Design 3 and Design 4.

To compute diversity gain [30], one must first compare a diversity antenna system to its equivalent single diversity antenna system in a given channel and analyze the difference in signal-to-noise ratio between the two. Based on Equation (3), it is possible to evaluate an envelope correlation coefficient and DG in terms of a theoretical maximum diversity gain of 10 dB. Together, the two aspects can be evaluated. Multi-user MIMO antenna patches get increasingly isolated as diversity gain rises. Therefore, DG should be at least 9 dB louder than that to restate the original question: DG values are expected to exceed 10 dB in all operational bands of both antenna, as seen in Figure 4. It ensures that the suggested MIMO structure’s diversity performance is sufficient.

**Figure 5:**
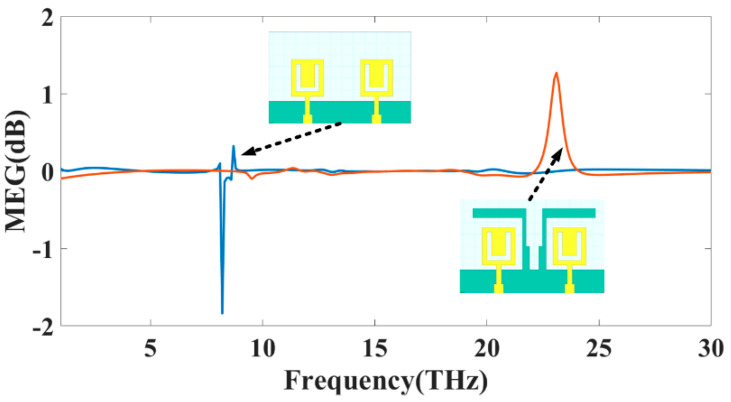
The MEG (dB) simulated value for MIMO structure Design 3 and 4.

According to the MEG parameter, the sum of the average power received by two isotropic antennas when there is no background noise is equal to or less than the average power received by a diversity antenna when noise is present (or interference). It shows how the surroundings affect a MIMO antenna’s improved performance. According to the equation suggested in [31], the presence of the MEG may be confirmed by using Equation (4). In this equation, *M* represents the MIMO design of a total number of ports, and radiation efficiency defined as $\eta_{i,\;\mathrm{rad}}$ of the current MIMO design structure. MEG should be set to −3 dB to provide the best possible diversity performance at each of the device’s ports. Additionally, the two ports must have a differential of no more than 0 dB. Figure 5 depicts the MEG < −3 dB values for each of the three operating modes.

**Figure 6:**
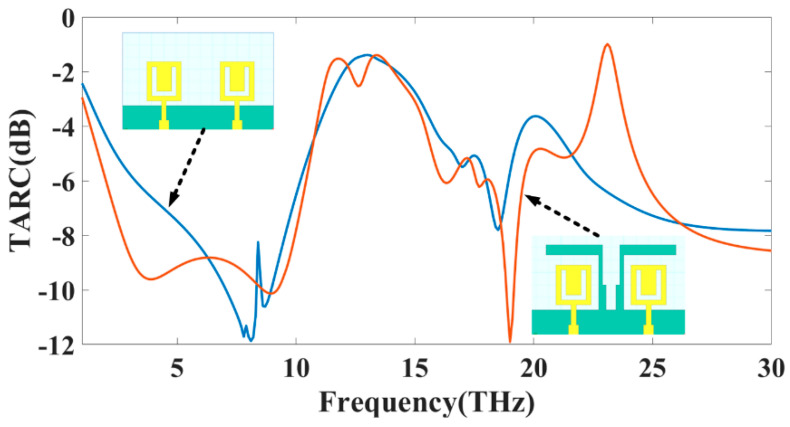
The simulated value of the TARC (dB) for MIMO structure Design 3 and Design 4.

The Total Active Reflection Coefficient, often known as TARC, is the most accurate method for quantifying radiation performance and frequency response when applied to more than one port. To compute it, take the square root of the total reflected power and divide that number by the total power that was incident to the object. TARC, which stands for “Total Active Reflection Coefficient”, is the method that is used to evaluate how effectively a MIMO system can bend light. This method considers the random signal pairings on the network and the mutual coupling on the network. We can observe how reflected and incident waves are characterized using Equation (5), which we can see below. It is possible to get this information by utilizing Equation (6) expressed in terms of S-parameters, as stated in [32]. The disparity in TARC values that results from the two distinct designs of the proposed MIMO antenna is seen in Figure 6. It was determined that the performance of the data acquired in the THz band using MIMO is suitable for the applications planned to use it.

(5) $$\Gamma_{a}^{t}=\frac{\sqrt{\Sigma_{j}^{M}{\left|b_{j}\right|}^{2}}}{\sqrt{\sum_{j}^{M}{\left|a_{j}\right|}^{2}}}$$

(6) $$\Gamma_{a}^{t}=\sqrt{\frac{{\left|S_{11}+S_{12}e^{j\theta }\right|}^{2}+{\left|S_{21}+S_{22}e^{j\theta }\right|}^{2}}{2}}$$

**Figure 7:**
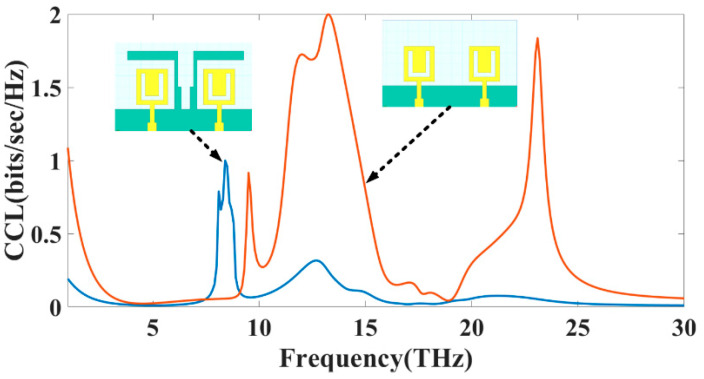
The simulated value of the CCL (dB) for MIMO structure Design 3 and Design 4.

The Channel Capacity Loss (CCL) is an additional crucial component that must be considered to evaluate the MIMO performance of the chosen THz antenna. The channel capacity loss determines the maximum pace at which information may be sent over the channel without suffering a significant loss. The rate must be lower than 0.5 bits/s/Hz to show information transmission without loss using a MIMO system that was effectively built. Using Equations (7)–(9) described in [32], it is feasible to compute the CCL parameter. According to the findings in Figure 7, this CCL limit was also attained for the multiple bands for both designs.

(7) $$C_{\mathrm{loss}\;}=-\log_{2}det\left(a^{R}\right)$$

(8) $$a^{R}=\left(\begin{array}{c} \rho_{11}\;\rho_{12}\\ \rho_{21}\;\;\rho_{22} \end{array}\right)$$

(9) $$\rho_{ii}=1-\left({\left|S_{ii}\right|}^{2}+{\left|S_{ij}\right|}^{2}\right),\;\mathrm{and}\;\rho_{ij}=-\left(s_{ii}^{*}S_{ij}+s_{ij}^{*}S_{ij}\right),\;\mathrm{where}\;i,j=1\;\mathrm{or}\;2$$

**Figure 8:**
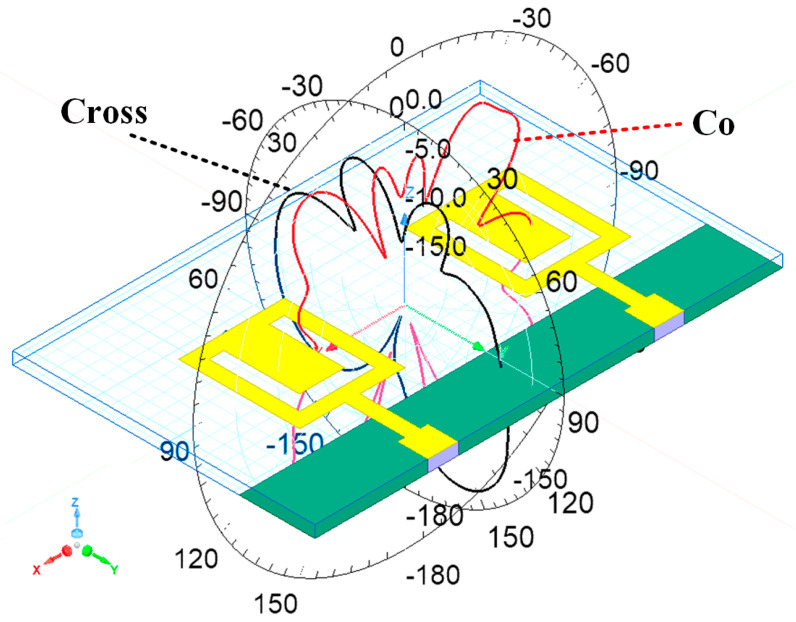
Graphical representation of the Co and cross-polarization field for the Hilbert-shaped antenna with its radiation pattern.

**Figure 9:**
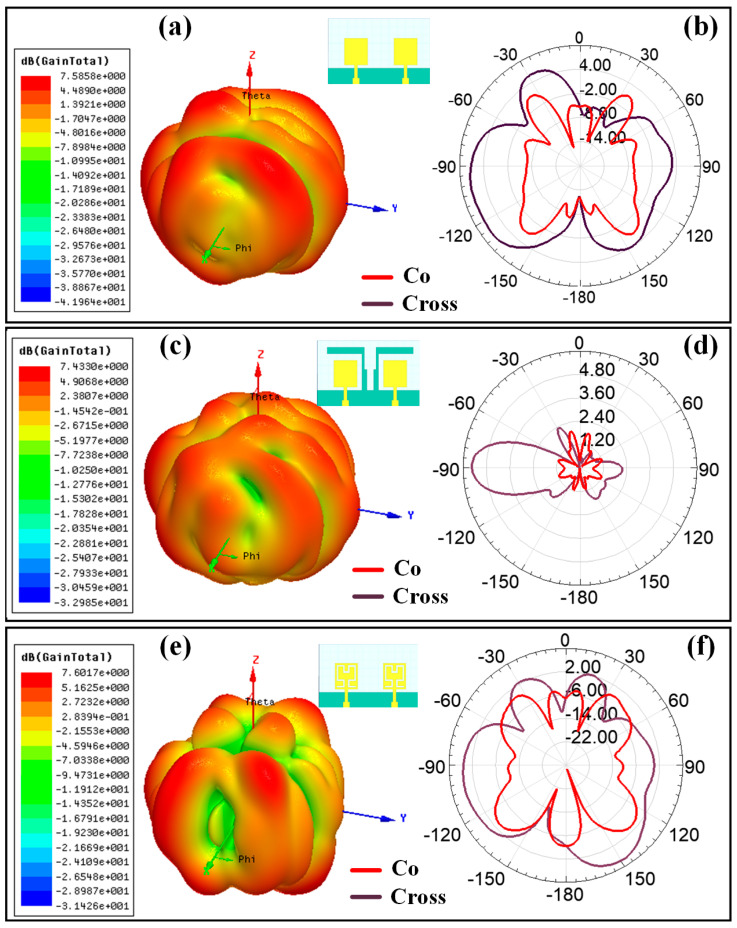
Polar plot for the different antenna structures (simulated results); 3D polar plot for (**a**) Design 1, (**c**) Design 2, and (**e**) Design 5. 2D polar plot for the (**b**) Design 1, (**d**) Design 2, and (**f**) Design 5 with co-polar and cross-polar response.

**Figure 10:**
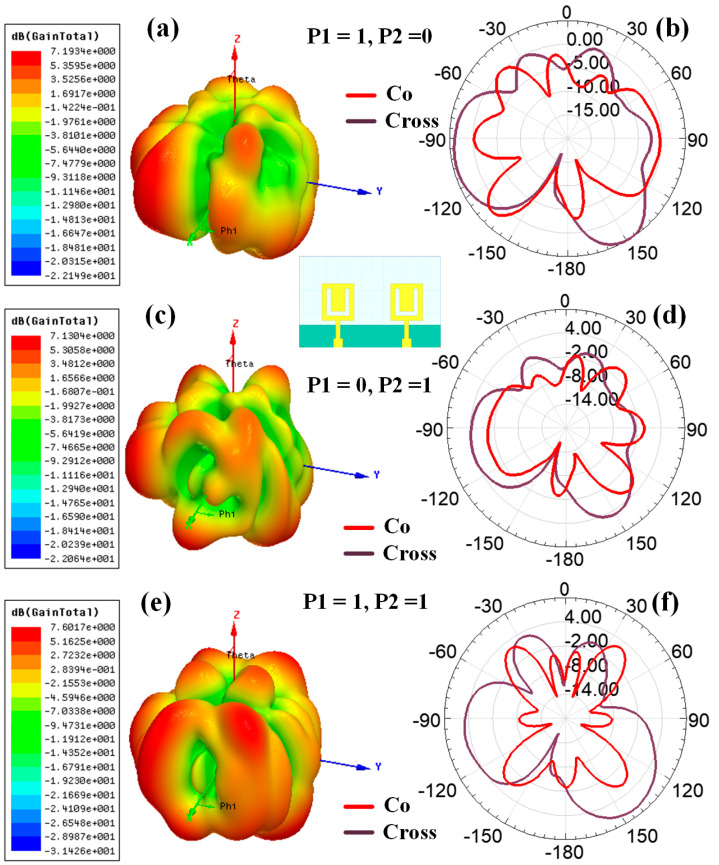
Variation in the polar plot for the different port excitation conditions (Design 3) (simulated results). The 3D polar plot for the (**a**) (P1, P2) = (1, 0), (**c**) (P1, P2) = (0, 1), and (**e**) (P1, P2) = (1, 1). 2D polar plot for the (**b**) (P1, P2) = (1, 0), (**d**) (P1, P2) = (0, 1), and (**f**) (P1, P2) = (1, 1), with co-polar and cross-polar conditions.

**Figure 11:**
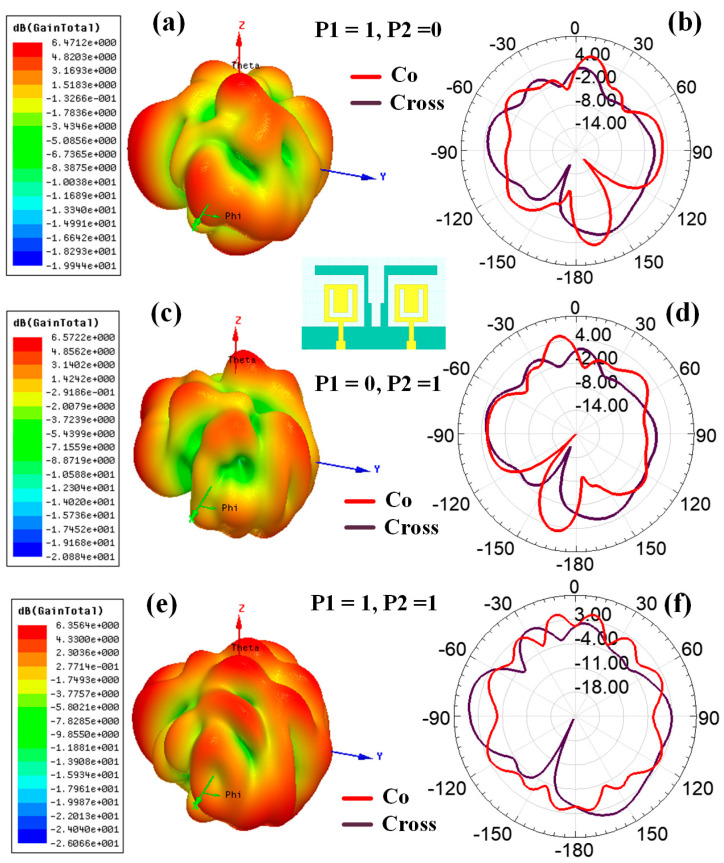
Variation in the polar plot for the different port excitation conditions (Design 4) (simulated results). The 3D polar plot for the (**a**) (P1, P2) = (1, 0), (**c**) (P1, P2) = (0, 1), and (**e**) (P1, P2) = (1, 1). 2D polar plot for the (**b**) (P1, P2) = (1, 0), (**d**) (P1, P2) = (0, 1), and (**f**) (P1, P2) = (1, 1), with co-polar and cross-polar conditions.

Figure 8 shows the graphical view of the co- and cross-polarization plot of the radiation pattern for the Hilbert shape antenna to identify the radiation plane. Figure 9 shows the variation in polarization for the different design structures. Figure 9a,c,e shows the variation in the 3D polar plot for designs 1, 2, and 5, respectively. Similarly, Figure 9b,d,f shows the variation in the 2D polar plots with co- and cross-polarization conditions for designs 1, 2, and 5, respectively. The maximum gain of 7.58 dBi, 7.43 dBi, and 7.60 dBi are shown for Design 1, Design 2, and Design 5, respectively. Figure 10 and Figure 11 show the variation in a polar plot for the different port excitation conditions. Figure 10a,b shows the polar plot for single port excitation, while Figure 10c shows the variation in the polar plot for both port excitation Design 3 structures. Similarly, Figure 11a,b shows the polar plot for single port excitation, while Figure 11c shows the variation in a polar plot for both port excitation Design 4 structures. It is plain to see that a single port or several ports of excitation, depending on the requirements of the particular application, can make a difference in how the polar field is influenced.

**Figure 12:**
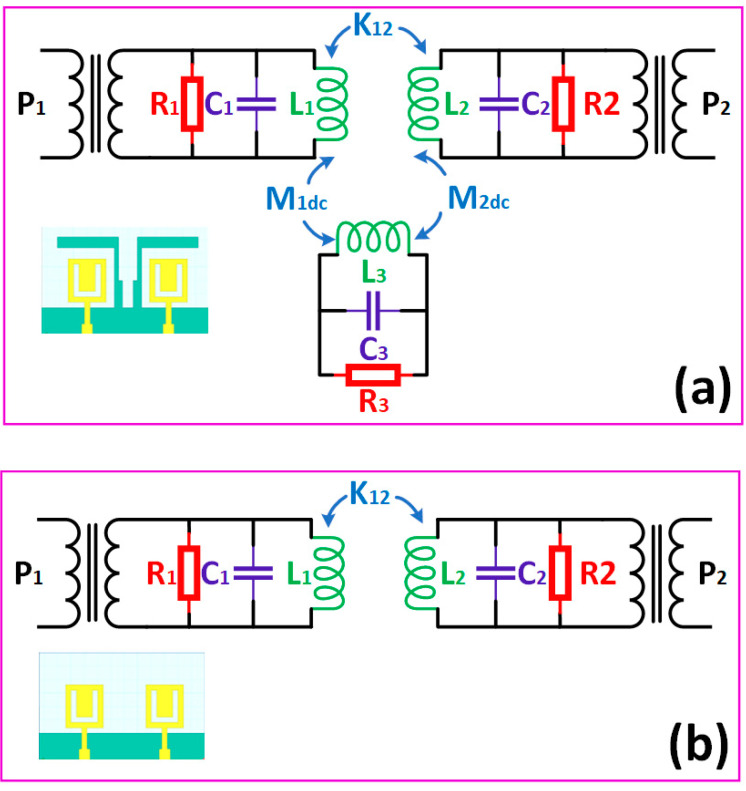
Possible equivalent RLC model for (**a**) Design 4 and (**b**) Design 3 structure of the proposed antenna.

**Figure 13:**
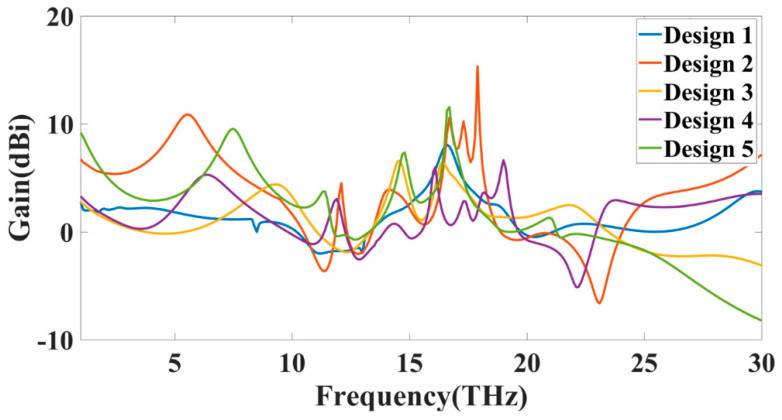
Calculated (simulated results) variation in gain (dBi) for all the antenna structures.

**Figure 14:**
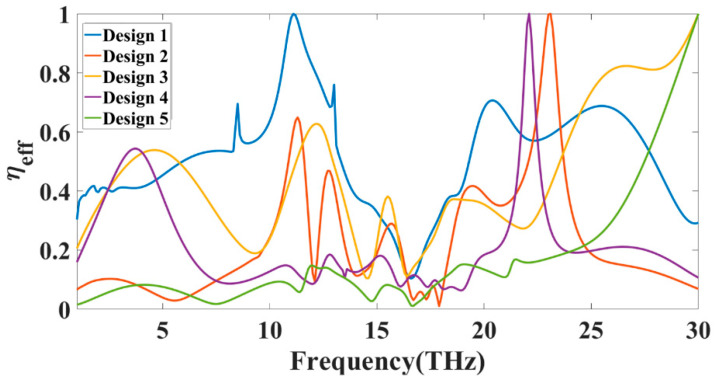
Calculated (simulated results) antenna efficiency for all the antenna structures for 1 to 30 THz of the frequency.

Figure 12 shows the proposed structure’s possible equivalent circuit model with RLC components. Many works suggest this RLC equivalent model for MIMO antennas [33,34,35,36]. Figure 12a,b shows the possible RLC model for Design 4 and Design 3. Design 4 contains the stub in the middle of the two radiators, which generates the mutual RLC component, as shown in Figure 12a. While in normal radiation, the mutual induction values can be represented as shown in Figure 12b. The overall gain achieved over the 1 to 30 THz frequency band for all the antenna structures is shown in Figure 13. The maximum gain is ~10 dBi for the majority of the first operation band < 15 THz frequency. The maximum gain for the >15 THz frequency is observed up to 17 dBi. Most antennas in these operating bands show a return loss of <−10 dB. Figure 14 shows the antenna radiation efficiency for all antenna structures. Designs 1, 3, and 4 show a higher radiation efficiency for <15 THz operating bands. Similarly, Designs 1, 2, 3, and 4 show a higher radiation efficiency for >15 THz operating bands.

**Figure 15:**
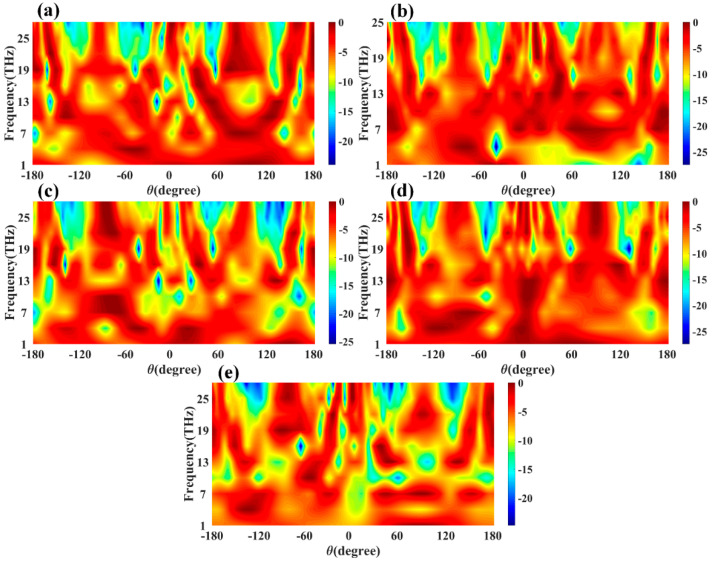
Calculated normalized gain variation for the frequency and theta (degree) variation. The contour plot of gain variation is plotted for (**a**) Design 1, (**b**) Design 2, (**c**) Design 3, (**d**) Design 4, and (**e**) Design 5 of the proposed antenna structures.

The efficiency shown in Figure 14 will rise for a specific frequency range where the return loss is also >−10 dB. These bands are considered the non-radiation band of the proposed antenna, where the antenna shows oscillating behaviour. Figure 15 shows the variation in the normalized gain for the different antenna designs. We can see the variation in the gain for the different values of the frequency and radiation angle theta. We can also find the half-power beam width from these plots, allowing us to choose the maximum peak gains for specific frequency and radiation angle ranges.

**Figure 16:**
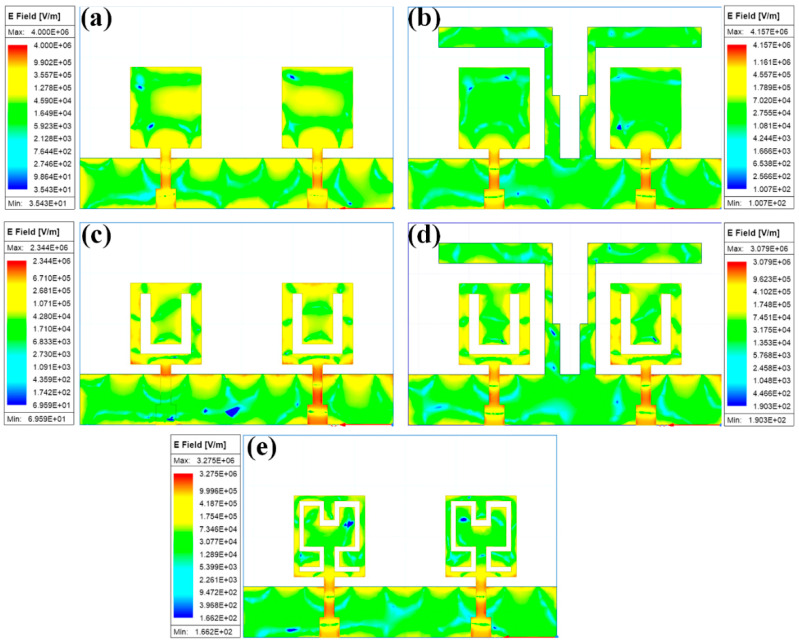
Normalized electric field distribution for the different (**a**) Design 1, (**b**) Design 2, (**c**) Design 3, (**d**) Design 4, and (**e**) Design 5 structures of the proposed antenna.

Figure 16 shows the normalized electric field intensity for the different antenna design structures. It is observed that the significant electric field coupling between two MIMO antenna elements is because of the ground patch and stub structure. We observed that the coupling between both antennas in the proposed structure changes the operating bands and effective return loss. The field distribution between two MIMO antennas is different in the simple, grounded patch (Figure 16a,c,e) and stub-based grounded patch (Figure 16b,d). The effective field distribution is also low in normal ground patch conditions compared to stub conditions. The concentrated electric field values in normal patch conditions of Designs 1 and 2 are relatively higher than the Hilbert-shaped structure. Table 4 presents the results of a comparison study conducted between the suggested MIMO antenna design and another type of THz antenna design regarding the dimensions, gain, bandwidth, operating band, efficiency, and substrate material. It was discovered that the presented antenna provides a broad spectrum of operation with a low return loss. The proposed antenna can be used in various industrial and medical applications. The lower THz range < 3 THz frequency range is applicable for different spectroscopy applications to detect chemical and biological substances in sealed packets or concealed in clothing. Since high-sensitivity coherent detection systems are currently available, THz radiation can be employed at microwatt power levels while still being safe for humans and other non-living things. This quality of THz radiation makes it applicable in biological and medical applications [37,38], such as medical imaging to detect infected tissues.

## 5. Conclusions

A graphene-based Hilbert-shaped MIMO antenna with multi-band characteristics is numerically investigated over 1–30 THz of the frequency range. We analyzed this antenna structure in different shapes to identify the influence on the MIMO antenna parameters, such as radiation response, return loss, number of operating bands, bandwidth, TARC, ECC, CCL, MEG, and DG. These antennas offer a minimum of three operating bands with a return loss of less than 10 dB. The maximum bandwidth offered by this antenna is 8.8 THz. The proposed antenna was measured to have a minimum return loss of −40.46 dB. The maximum gain is observed as ~10 dBi for the band of <15 THz and ~17 dBi for the band of a >15 THz frequency range of the proposed antenna structures. The different port excitation conditions can modify the polar response of the antenna. Within this range, all of the requirements for the MIMO antenna values were met for successful radiation operation. Consequently, this Multiple-Input Multiple-Output (MIMO) antenna is appropriate for use in applications requiring high-speed operation within the THz frequency range and taking place within a small radius inside.

## Figures and Tables

**Table 1:** Dimensions of the antenna (in µm).

Dimensions	L	W	w	m	s	p	q	a	b	c	d	e	f	g	h
Value	620	400	40	225	160	100	30	40	20	30	50	140	160	15	100

**Table 2:** Different antenna designs and their specifications.

Design	Structure	Specification
Design 1	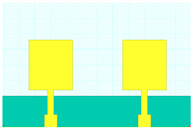	Simple patch antenna with the rectangular backside.
Design 2	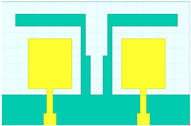	Simple patch antenna with modified back side with covered sides.
Design 3	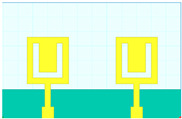	Hilbert shape order 1 with the rectangular back side.
Design 4	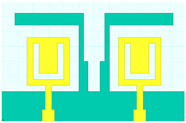	Hilbert shape order 1 with modified back side with covered sides.
Design 5	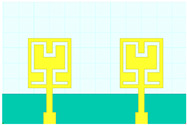	Hilbert shape order 2 with the rectangular back side.

**Table 3:** Comparative analysis of all the designs regarding return loss, number of operating bands, and bandwidth.

Design	Number of Band	Minimum Return Loss (dB)	$f_{min}$ (THz)	$f_{max}$ (THz)	Bandwidth $f_{max}\;$$-\;f_{min}$ (THz)	MIMO Order	Antenna Efficiency	Maximum Gain (dBi)
Design 1	1	−26.58	2.1	7.6	5.5	0	0.53	2.5
	2	−10.23	9	9.5	0.5		0.57	1.6
	3	−39.059	15.6	19.8	4.2		0.64	9.2
	4	−16.67	23.7	30	6.3		0.68	4.5
Design 2	1	−22.91	2	9.4	7.4	0	0.17	11.3
	2	−11.38	9.6	10.5	0.9		0.25	5.3
	3	−38.09	15.7	18.9	3.2		0.36	16.2
	4	−17.46	24.4	30	5.6		0.25	7.1
Design 3	1	−40.46	3.3	9.8	6.5	1	0.53	5.1
	2	−29.57	14.1	20	5.9		0.38	7.3
	3	−12.39	24.9	30	5.1		0.99	0.1
Design 4	1	−29.78	3.6	10.5	6.9	1	0.54	5.5
	2	−34.24	15.5	20.3	4.8		0.19	5.9
	3	−15.87	24.2	30	5.76		0.21	4.4
Design 5	1	−24.7	1.3	10.1	8.8	2	0.1	9.8
	2	−25.61	14.8	19.3	4.5		0.15	11.3
	3	−15.51	23.3	28.2	4.9		0.68	0.2

**Table 4:** Comparative analysis of the proposed structure with previously published articles.

References	Dimension (µm^2^)	Operating Frequency	Gain (dBi)	Bandwidth (THz)	Substrate	Return Loss (dB)	Radiation Efficiency
This design	620 × 400 µm^2^	1–30 THz	~10	8.1	polyimide	−40.46	99%
[39]	500 × 500 µm^2^	0.5 −1 THz	10.7	9.8	Photonic crystal	−35	79.7%
[40]	500 × 500 µm^2^	0.5–0.7 THz	7.3	-	Pyrex	−18	-
[41]	1000 × 1000 µm^2^	0.5–0.85 THz	3.52	0.15	RT/Duroid 6006	−42	55.85%
[42]	400 × 400 µm^2^	0.7–1.1 THz	10.45	0.119	Triethylamine	−27	90.69%
[43]	109.76 × 150.93 µm^2^	0.5–1 THz	-	0.13	Tetrafluoroethylene	−32	-
[44]	600 × 700 µm^2^	0.7–0.85 THz	9.7	0.15	RT/Duroid 6006	−52	75%
[45]	300 × 300 µm^2^	0.35–0.75 THz	5.7	0.269	Polyimide	−17	97.3%
[46]	1000 × 1000 µm^2^	0.63–0.8 THz	10.43	0.155	RT/Duroid 6006	−41	-
[47]	600 × 800 µm^2^	0.6–0.7 THz	8	0.0364	Polyimide	−45	-
[48]	600 × 600 µm^2^	0.51–0.78 THz	9.19	0.2	Polyimide	−57	90.84%

## Data Availability

Data available based upon reasonable request from corresponding author.
